# Treatment of bipolar disorder in the Netherlands and concordance with treatment guidelines: study protocol of an observational, longitudinal study on naturalistic treatment of bipolar disorder in everyday clinical practice

**DOI:** 10.1186/1471-244X-14-58

**Published:** 2014-02-28

**Authors:** Joannes W Renes, Eline J Regeer, Trijntje YG van der Voort, Willem A Nolen, Ralph W Kupka

**Affiliations:** 1 Altrecht Institute for Mental Health Care, Lange Nieuwstraat 119, 3512 PG Utrecht, The Netherlands; 2 Department of Psychiatry, VU University Medical Center, A.J. Ernststraat 1187, 1081 HL Amsterdam, The Netherlands; 3 Department of Psychiatry, University Medical Center, University of Groningen, Hanzeplein 1, 9713 GZ Groningen, The Netherlands

**Keywords:** Bipolar disorder, Clinical practice, Guidelines, Care as usual

## Abstract

**Background:**

While various guidelines on the treatment of bipolar disorder have been published over the last decades, adherence to guidelines has been reported to be low. In this article we describe the protocol of a nationwide, multicenter, longitudinal, non-intervention study on the treatment of bipolar disorder in the Netherlands. Study aims are to provide information on the nature and content of outpatient treatment of bipolar disorder, to determine to what extent treatment is in concordance with the Dutch guideline for the treatment of bipolar disorder (2008), and to investigate the relationship of guideline concordance with symptomatic and functional outcome.

**Methods/Design:**

Between December 2009 and February 2010, all psychiatrists registered as member of the Dutch Psychiatric Association received a questionnaire with questions about their treatment setting, and whether they would be willing to participate in further research. Psychiatrists treating adult outpatients with bipolar disorder were invited to participate. Consenting psychiatrist subsequently approached all their patients with bipolar disorder. The study is performed with written patient and caregiver surveys at baseline and after 12 months, including data on demographics, illness characteristics, organization of care, treatments received, symptomatic and functional outcome, quality of life, and burden of care for informal caregivers.

**Discussion:**

This study will provide information on the naturalistic treatment of bipolar disorder in the Netherlands, as well as degree of concordance of this treatment with the Dutch guideline, and its relationship with symptomatic and functional outcome. Limitations of a survey-based study are discussed.

## Background

Bipolar disorder is a severe mental disorder characterized by recurrent episodes of mania, hypomania, depression, or mixed episodes. The estimated lifetime prevalence of bipolar disorder ranges from 1,5 to 2% [1]. Many patients with bipolar disorder do not regain full psychosocial functioning between episodes and health related quality of life is adversely affected for many patients [2,3]. Although patients themselves are most affected by the illness, informal caregivers also report distress and have difficulties in coping with the illness [4].

To improve quality of care, various guidelines on the treatment of bipolar disorders have been published over the last decades. Recent studies on collaborative care programs for bipolar disorders [5-8] show that patients treated in speciality programs (consisting of an intensive follow-up by a psychiatric nurse, a psycho-education program, algorithm-based advices on medication treatments for the treating psychiatrist and an “emergency plan” on how to deal with manic and depressive symptoms) have less manic symptoms and spent less time in manic episodes compared with patient treated with care as usual. Patients also report better quality of life and improved social functioning. Recently, Kessing et al. [9] showed that patients with bipolar disorder in the early course of their illness had a more favorable outcome when treated in a specialized mood disorder clinic versus standard outpatient treatment. As was shown in the Texas Medication Algorithm Project [10] patients with bipolar disorder show a significant improvement in psychiatric symptoms when medication algorithms are implemented in their treatment. It can be assumed that when patients are treated according to guidelines, quality of care as well as treatment outcome will improve.

Although little research has been done on adherence to guidelines for the treatment of bipolar disorder in daily practice [11], there is some evidence that without specific interventions adherence to guidelines by care providers is low.

Using data from a U.S. medical benefits database, Baldessarini et al. [12] reported that only a quarter of 7760 patients with bipolar disorder in a large community sample was using a mood stabilizer as initial monotherapy. A retrospective study among 1471 bipolar I patients who were discharged from hospital showed that when patients were admitted because of a manic or depressive episode, treatment followed guidelines in patients without psychotic features in only 16% and 17% respectively [13]. Percentages were higher in patients with psychotic features but still only 38% for patients with psychotic mania and 31% for patients with psychotic bipolar depression. Using data from the National Ambulatory Medical Care Survey, which is conducted annually by the National Center for Health Statistics in the U.S. and samples a representative group of visits to physicians in office-based practices, Blanco et al. [14] analysed medication treatment in 865 visits to a psychiatrist by patients with a diagnosis of bipolar disorder. Also in this study, mood stabilizers were underused.

As expected, treatment in university and tertiary centers had greater concordance with guidelines. Of the first 500 patients entering the U.S. Systematic Treatment Enhancement Program for Bipolar Disorder (STEP-BD), 72% were taking a mood stabilizer [15]. Although certain multifaceted and resource-intensive interventions in research settings improve adherence to treatment guidelines, after cessation of these interventions, adherence rates returned to pre-intervention levels [11].

To improve our knowledge on the implementation of treatment guidelines for bipolar disorder in daily clinical practice, and the relationship of concordance with guidelines with clinical and other outcomes, further studies are warranted. In 2008, a revised guideline for the diagnosis and treatment of bipolar disorder was published by the Dutch Psychiatric Association [16].

The study described in this article is an ongoing nation-wide study in the Netherlands among patients with bipolar disorder or schizoaffective disorder, their treating psychiatrists, and important significant others of participating patients. The purpose of the current study is to investigate current practice for patients with bipolar disorder or schizoaffective disorder in various treatment settings in the Netherlands, to assess concordance of provided treatments with the Dutch guideline and to assess the relationship of concordance with the guideline with symptomatic and functional outcome, quality of life, and satisfaction with care as reported by patients, and with the burden of care as perceived by significant others. Since the care recommended in the Dutch guideline for bipolar disorder resembles the previous mentioned speciality programs in many ways, concordance with the guideline is expected to improve quality of care and favor treatment outcome. In addition, factors that influence concordance with the guideline will also be investigated.

## Methods/Design

### Study design

This is a non-interventional, multicenter study with one year follow-up among patients with bipolar I disorder, bipolar II disorder, bipolar disorder not otherwise specified, or schizoaffective disorder, bipolar type, age 18 years and older. Patients with these disorders are included in the study since the Dutch guideline for bipolar disorder applies to these diagnostic categories. Since the purpose of the study is to provide valid information on naturalistic treatment in every day clinical practice and to investigate concordance with the current Dutch guideline for the treatment of bipolar disorder, the influence of the study on ongoing treatments should be as minimal as possible. To collect information on every day clinical practice, psychiatrists and patients from a wide range of treatment settings including non-specialised centers are asked to participate in the study. To minimize the effort to participate, the study is carried out with written surveys only, at two consecutive moments in time. Participating psychiatrists receive a survey about the organisation of care for patients with bipolar disorder in their treatment center or private practice at study entry and one year later. At baseline they are asked to invite all their eligible patients to participate in the study. At study entry and at one year follow-up patients receive two surveys, one for themselves and one for a significant other (informal caregiver). The survey for patients includes questions about illness characteristics, treatments received in the prior 12 months, and outcome measurements. Although 12 months follow-up may seem long and may cause some recall bias, 12-months follow-up is chosen because patients who are doing well may receive no care within a shorter duration of follow-up. And since one of the aims of the study is to investigate whether improvement in concordance with the guideline over time may lead to better quality of care and treatment outcome, enough time between measurements is needed to make changes in the care provided possible. Significant others receive a questionnaire about the burden of care. Information about individual treatments is given by the patients, since it is assumed that patients can report more accurately on the treatments they receive than treating health care providers, and to reduce the burden to participate for the latter. Moreover, health care providers may not be aware of whether patients are compliant or not. Also when this information is provide by health care providers it may influence the care they provide, and subsequently influence concordance with the guideline.

### Ethical considerations

The study protocol was approved by the Medical Ethical Committee of the University Medical Center Utrecht, The Netherlands, and was in addition independently reviewed by the scientific committees of the two main research centers, Altrecht Institute for Mental Health Care, Utrecht, The Netherlands, and GGZ inGeest/VU University Medical Center, Amsterdam, The Netherlands. All participating patients gave informed consent. Data are stored in a research database in accordance with the Dutch Data Protection Authority.

### Study population

Since the long-term treatment of bipolar disorder mainly takes place in outpatient settings, only patients treated there are included in the study. In the Netherlands general practitioners mostly refer patients with bipolar disorder to psychiatric treatment settings (mental health institutions, psychiatric departments of general hospitals, academic centers, or private practices). Therefore general practitioners and bipolar patients treated only by general practitioners are not included in the study. To be able to include psychiatrists and patients from a wide range of treatment settings, an exploratory survey was held prior to the start of the current study, between December 2009 and February 2010, among all 2525 psychiatrists who are member of the Dutch Psychiatric Association. All members were approached with a 5-item questionnaire about their treatment setting, the number of patients with bipolar disorder they treat, and whether they would be willing to participate in further research on the treatment of bipolar disorder. Reminders were sent to all non-responding psychiatrists. Of the 2525 psychiatrists 1579 (62.5%) responded of whom 616 (24.4%) were willing to participate in further research. Participating psychiatrists identified all eligible patients with a bipolar or schizoaffective disorder. Only patients unable to read Dutch or understand the questionnaires were excluded from participation in the study. There are no other exclusion criteria. The study is planned to end mid 2014.

### Methods of inclusion

Of the psychiatrists who responded to the explorative survey and were willing to participate in further research, five hundred and forty (21.4%) were treating adult patients with bipolar disorder, and were subsequently invited to participate in the current study. They received the baseline survey with more detailed questions about their treatment setting (including degree of specialisation in the treatment of mood disorders, composition of treatment staff, education on bipolar disorder of health care providers, and use of disorder-specific outcome measurements), and the number of patients potentially eligible for the study. Psychiatrists (N = 65) only treating children with bipolar disorder were not included. Psychiatrists who returned the survey subsequently received envelopes with information about the study and an informed consent form, to send to all eligible patients. Patients were only included in the study after the investigators had received a signed informed consent form. Patients then received the surveys, to be returned to the investigators. Since it was anticipated that only a proportion of eligible patients would participate, psychiatrists were asked to keep a record including information on age, gender, and type of bipolar disorder of all patients who would be invited. It will then be possible to compare participating patients with eligible patients who did not participate to make an estimate of the generalizability of the results. Of all participating patients a DSM-IV-TR axes I and II classification is obtained from the treating psychiatrists.

### Measurements

Measurements take place at baseline and at 12 month follow-up in patients, psychiatrists and significant others (Table 1). Since the study is carried out with surveys only, some scales were adapted to serve as self-administrated instruments. The following outcome measurements are collected at both time points. *Global clinical outcome*: a modified self rated version of the Clinical Global Impression scale for Bipolar disorder (CGI-BP). The CGI-BP measures global symptom severity and impact on functioning, as well as change over time. We used only the scale for change, i.e., improvement or worsening of symptoms and functioning in comparison with 12 month before. Although the original clinician-rated CGI-BP-change has been validated [17], there are currently no data available on a self-rating version that we are aware of. A problem with a self-rating version may be that patients may overestimate or underestimate symptoms and functioning in comparison with a clinician rating.

**Table 1:** Study plan and assessments

	**Baseline**	**12 month follow-up**	
Demographics	x		Cultural background, marital status, gender, age, level and total years of education, and professional status
Illness characteristics	x		Age of onset, total number of episodes and admissions, suicide attempts, severity of mood symptoms, family history of psychiatric disorders, substance abuse
Treatment information	x	x	Health care providers involved, agreement on treatment plan, follow-up patterns, type of maintenance pharmacotherapy, participation in group and non-drug therapies (psycho-education, use of an emergency plan and prospective lifechart, social rhythm and other psychotherapies)
Clinical outcome	x	x	CGI-BP-change (modified), QIDS-SR, ASRM
Satisfaction with care	x	x	10 point Likert scale
Quality of life	x	x	WHOQoL-bref
Functioning	x	x	FAST (modified)
Adherence	x	x	DAI-10
Burden of care	x	x	BES
Treatment center information	x	x	Treatment setting, composition of treatment staff, availability of group psychoeducation program, use of rating scales, additional training in the treatment of bipolar disorder (e.g. attendance of conferences, training programs), number of patients with bipolar disorder treated

*Severity of symptoms*: the Quick Inventory of Depressive Symptomatology (QIDS-SR) for assessment of depressive symptoms, a 16-item self-administrated rating scale with good psychometric properties [18]. The Altman Self-Rating Mania Scale (ASRM), a self-administrated rating scale for the assessment of manic symptoms. This scale has been validated in international studies [19]. *Satisfaction with care*: scoring by patients on satisfaction of the treatment received with a scoring between 0 (very unsatisfied, worst possible treatment) and 10 (very satisfied, best possible treatment). *Quality of life*: the WHOQoL-bref, a short version (26 items) of the original 100 items scale developed by the WHO for the measurement of health related quality of life. The Dutch version was studied in 533 patients [20].

*Functioning*: a modified self-rated version of the Functioning Assessment Short Test (FAST), a brief instrument designed to assess the main functioning problems experienced by psychiatric patients, particularly bipolar patients; the clinician-rated FAST was described and validated by Rosa et al. [21]. There is discussion on patients’ ability to score quality of life or functioning on self-rating scales, as it may be biased by mood state. Given the design of this study it was not possible to implement clinicians rating scales.

*Adherence to treatment*: patients’ subjective sensations or beliefs with medication is measured with the Drug Attitude Inventory (DAI-10). The DAI-10 is originally designed to discriminate the compliance rate in schizophrenic patients, but can be used in other psychiatric disorders as well [22]. Patients are asked to their opinion with a true or false answer on 10 statements concerning the use of medication.

*Burden of care*: the “Betrokkenen Evaluatie Schaal” (BES), a scale for the assessment of consequences for caregivers of patients with severe mental illness, developed and validated in the Netherlands by van Wijngaarden et al. [23].

### Concordance with Dutch guideline for bipolar disorder

The Dutch guideline for the diagnosis and treatment of patients with bipolar disorders (further referred to as: “the guideline”) distinguishes between treatment modalities as recommended/obligatory for all patients (pharmacotherapy, providing information about the illness and the treatment alternatives, participation in a psychoeducation program, and interventions aimed at improving self-management) and as optional only for specific patient groups (psychotherapy and supportive treatment with rehabilitation interventions based on assessment of needs).

Since this study is performed with surveys only, there are some limitations in the assessment of whether certain treatment modalities were applied. For example, regarding individual psychoeducation and self-management, which includes a wide variety of interventions, obtaining standardized information is less feasible.

Concordance with the guideline will be assessed for the following treatment modalities (Table 2). *Psychoeducation*: taking part in a group psychoeducation program is recommended for all patients. *Use of an emergency plan on how to deal with early symptoms of a new mood episode* is considered an important self-management tool and its use is recommended for unstable patients, although instability of mood is not further specified in the guideline. We consider patients to be unstable when at least one mood episode occurred in the previous 12 months, being currently symptomatic, or requiring more than four visits with a mental health care provider in the past year. The latter is based on the assumption that in the Netherlands most patients without comorbid disorders will not have more than four visits a year with a mental health care provider when their mood is stable. The frequency of four visits per year is based on the fact that in the guideline measurement of lithium serum levels is recommended at 3–6 months intervals and that prescription of medication is allowed for a maximum duration of three months.

**Table 2:** **Criteria for concordance with the Dutch guideline for diagnosis and treatment of bipolar disorder**[16]

**Treatment modalities**	**Criteria for concordance with the Dutch guideline**
Group psycho-education^a^	All patients
Emergency plan on how to deal with emerging mood symptoms	All instable patients. (A mood episode in the previous 12 month, currently symptomatic, more than four visits a year)
Maintenance pharmacotherapy	Patients with three or more episodes lifetime or a history with one or two severe episodes and/or a positive family history of bipolar disorder
Use of prospective lifechart	Rapid cycling, a mood episode in the previous 12 months or currently symptomatic despite maintenance pharmacotherapy
Specific psychotherapy for bipolar disorder and/or supportive treatment	Instability despite medication and participation in a group psychoeducation program

^a^In the Netherlands a group psycho-education program described by Hofman et al. [24] is advised for all patients. This program consists of 6 sessions for patients and caregivers.

*Maintenance pharmacotherapy*: maintenance pharmacotherapy is considered to be concordant with the guideline when medication recommended for maintenance therapy is used. In the guideline maintenance pharmacotherapy is indicated in all patients with three or more previous episodes. For patients with only one or two previous episodes, maintenance pharmacotherapy is indicated when at least one of the episodes was severe or when there is a first-degree relative with bipolar disorder.

*Use of a prospective lifechart*: in the guideline use of a prospective lifechart for continuous mood-monitoring is recommended for patients with mood instability (rapid cycling) or treatment resistance. Apart from rapid cycling in the past year, we consider a breakthrough mood episode in the previous 12 months or currently being symptomatic despite maintenance medication an indication for the use of a prospective lifechart. In the guideline *specific psychotherapy for bipolar disorder* and *supportive treatment* are recommended as optional treatment indicated for various patient groups.

Due to the study design, information on patients groups for which these optional modules would be indicated is however limited. To assess concordance with the guideline we therefore limited the recommendation for specific psychotherapy for bipolar disorder and/or supportive treatment to patients who are unstable despite medication and psychoeducation. Supportive treatment is defined by the participation of a mental health nurse in the treatment.

With these criteria for concordance with the guideline, three groups of patients can be distinguished requiring different treatment modalities to be minimally part of the treatment: 1. Patients without an indication for maintenance pharmacotherapy and currently stable, for which minimally participation in a group psychoeducation program is required, 2. Patients with an indication for maintenance pharmacotherapy and currently stable, requiring both participation in a group psychoeducation program and maintenance pharmacotherapy, and 3. patients currently unstable despite medication and psychoeducation, requiring all above mentioned treatment modalities.

### Factors influencing concordance with the guideline

One of the aims of this study is to investigate which factors are associated with concordance with the treatment guideline, in particular whether patients treated in specialized outpatient centers in the Netherlands receive treatment that is more in concordance with the guideline. Three degrees of specialization are distinguished. Centers treating patients from different diagnostic groups, centers treating patients with mood disorders and centers treating only patients with bipolar disorder. In addition to the degree of specialization of treatment centers, demographics, illness characteristics, provision of care by the same health care provider and agreement on treatment plan by the patients will be investigated as factors that influence concordance with the guideline.

### Statistical analysis

Descriptive statistics will be used and include frequency tables, (n, mean, median, standard deviations, minimum and maximum for continuous measures and n, frequencies and percentages for categorical measures). Two-sided 95% confidence intervals will be obtained. For the associations between patients’ characteristics, concordance with treatment guideline (as independent variable) and outcome measures, general and logistic models are planned. Odds ratios and 95% confidence intervals will be reported and P values for differences between groups for concordance with treatment guideline.

### Sample size

The main aim of the study is to describe outpatient care as usual for bipolar patients in the Dutch population. Therefore no minimal sample size was set prior to the study; inclusion procedures were designed to include as many as possible patients from as many different treatment settings as possible.

## Discussion

This is the first nationwide study on the quality of care for patients with bipolar disorder in the Netherlands. Like the large international non-interventional Wide AmbispectiVE study of the clinical management and burden of Bipolar Disorder (WAVE-bd) [25], this study will provide information on a wide variety of treatments in the clinical management of bipolar disorder.

So far data on naturalistic treatment of bipolar disorder in the Netherlands are mainly available from patients who participated in the multicenter multinational longitudinal study of the former Stanley Foundation Bipolar Network [26,27]. However, that study included patients who were motivated to participate in intensive longitudinal and interventional research, and thus may not be representative for the average outpatient. We therefore intended to create a minimal threshold for participation and no interference with ongoing treatment.

A second aim of the study is to assess concordance of treatments with the Dutch guideline. When investigating concordance with treatment guidelines in naturalistic treatment settings, a problem may be that participating health care providers may change their habits and start providing treatments which are more in concordance with guidelines. Although this may improve the quality of care for patients participating in a study, it may portray a more favorable outcome than will be the case in every day practice.

In this study we aim to minimize the influence on participating health care providers by using written surveys only and limit the measurements to only two moments in time. Moreover, information on treatment details and outcome is given by the patient, who will be less aware of the recommendations given in the guideline. Although we assume this will provide more valid information on current treatments, studies with written surveys also have limitations.

Non-response with possible selection bias may limit generalizability of research findings. To minimize selection bias we approached all eligible psychiatrists in the Netherlands and their patients to participate in the study. Another limitation that may occur with written surveys is recall bias. Patients may for instance have problems remembering visits to health care providers or medication used. We therefore limited data collection to the previous 12 month at both time points (with the exception of previous participation in a group psychoeducation program since patients may have participated in such a program more than a year ago prior to study entry).

Despite these limitations we expect that this study will contribute to the quality of care for patient with bipolar disorder by providing information on treatment of bipolar disorder in everyday clinical practice and the impact of concordance with the guideline. Insight in factors that are associated with concordance with the guideline will help to develop more effective strategies to implement evidence-based treatments in clinical practice. We think the design of the study is innovative since it combines information on everyday clinical practice, the impact of concordance with the guideline and the organization of care. Moreover concordance with the guideline will be assessed in different subgroups of patients requiring different treatment modalities as recommended in the guideline, instead of more general recommendations. We hope that with the publication of the methodology of our study, we will contribute to discussion on how studies on naturalistic treatments and concordance of treatment guidelines can be designed, helping to close the cap between evidence based treatments in guidelines and care in every day clinical practice.

## Competing interests

JWR received funding for this study by Altrecht Institute for Mental Health Care.

RWK received an unrestricted research grant for this study by AstraZeneca.

## Authors’ contributions

JWR drafted this paper and it was modified by all authors. WAN and RWK conceived the study, all othter authors, JWR, EJR and TYGV contributed to the design and study protocol. All authors approved the final manuscript.

## Pre-publication history

The pre-publication history for this paper can be accessed here:

http://www.biomedcentral.com/1471-244X/14/58/prepub

## References

[B1] Pini S de Queiroz V Pagnin D Pezawas L Angst J Cassano GB Wittchen HU Prevalence and burden of bipolar disorders in European countries Eur Neuropsychopharmacol 2005 15 425 434 10.1016/j.euroneuro.2005.04.011 15935623

[B2] IsHak WW Brown K Aye SS Kahloon M Mobaraki S Hanna R Health-related quality of life in bipolar disorder Bipolar Disord 2012 14 6 18 10.1111/j.1399-5618.2011.00969.x 22329468

[B3] MacQueen GM Young LT Joffe RT A review of psychosocial outcome in patients with bipolar disorder Acta Psychiatr Scand 2001 103 163 170 10.1034/j.1600-0447.2001.00059.x 11240572

[B4] Goossens PJ Van Wijngaarden B Knoppert-van Der Klein EA Van Achterberg T Family caregiving in bipolar disorder: caregiver consequences, caregiver coping styles, and caregiver distress Int J Soc Psychiatry 2008 54 303 316 10.1177/0020764008090284 18720891

[B5] Bauer MS The collaborative practice model for bipolar disorder: design and implementation in a multi-site randomized controlled trial Bipolar Disord 2001 3 233 244 10.1034/j.1399-5618.2001.30502.x 11903206

[B6] Bauer MS McBride L Williford WO Glick H Kinosian B Altshuler L Beresford T Kilbourne AM Sajatovic M Cooperative Studies Program 430 Study T Collaborative care for bipolar disorder: part I. Intervention and implementation in a randomized effectiveness trial Psychiatr Serv 2006 57 927 936 10.1176/appi.ps.57.7.927 16816276

[B7] Simon GE Ludman EJ Unutzer J Bauer MS Operskalski B Rutter C Randomized trial of a population-based care program for people with bipolar disorder Psychol Med 2005 35 13 24 10.1017/S0033291704002624 15842025

[B8] Simon GE Ludman EJ Bauer MS Unutzer J Operskalski B Long-term effectiveness and cost of a systematic care program for bipolar disorder Arch Gen Psychiatry 2006 63 500 508 10.1001/archpsyc.63.5.500 16651507

[B9] Kessing LV Hansen HV Hvenegaard A Christensen EM Dam H Gluud C Wetterslev J Early Intervention Affective Disorders Trial G Treatment in a specialised out-patient mood disorder clinic v. standard out-patient treatment in the early course of bipolar disorder: randomised clinical trial Br J Psychiatry 2013 202 212 219 10.1192/bjp.bp.112.113548 23349295

[B10] Suppes T Rush AJ Dennehy EB Crismon ML Kashner TM Toprac MG Carmody TJ Brown ES Biggs MM Shores-Wilson K Witte BP Trivedi MH Miller AL Altshuler KZ Shon SP Texas Medication Algorithm Project, phase 3 (TMAP-3): clinical results for patients with a history of mania J Clin Psychiatry 2003 64 370 382 10.4088/JCP.v64n0403 12716236

[B11] Bauer MS A review of quantitative studies of adherence to mental health clinical practice guidelines Harv Rev Psychiatry 2002 10 138 153 10.1080/10673220216217 12023929

[B12] Baldessarini RJ Leahy L Arcona S Gause D Zhang W Hennen J Patterns of psychotropic drug prescription for U.S. patients with diagnoses of bipolar disorders Psychiatr Serv 2007 58 85 91 10.1176/appi.ps.58.1.85 17215417

[B13] Lim PZ Tunis SL Edell WS Jensik SE Tohen M Medication prescribing patterns for patients with bipolar I disorder in hospital settings: adherence to published practice guidelines Bipolar Disord 2001 3 165 173 10.1034/j.1399-5618.2001.30401.x 11552955

[B14] Blanco C Laje G Olfson M Marcus SC Pincus HA Trends in the treatment of bipolar disorder by outpatient psychiatrists Am J Psychiatry 2002 159 1005 1010 10.1176/appi.ajp.159.6.1005 12042190

[B15] Ghaemi SN Hsu DJ Thase ME Wisniewski SR Nierenberg AA Miyahara S Sachs G Pharmacological treatment patterns at study entry for the first 500 STEP-BD participants Psychiatr Serv 2006 57 660 665 10.1176/appi.ps.57.5.660 16675760

[B16] Nolen WA Kupka RW PFJS der Klein EAM K-v Honig A Reichart CG Goossens PJJ Daemen P Ravelli DP Richtlijn Bipolaire stoornissen. Nederlandse Vereniging voor Psychiatrie 2008 Utrecht: De Tijdstroom

[B17] Spearing MK Post RM Leverich GS Brandt D Nolen W Modification of the Clinical Global Impressions (CGI) scale for use in bipolar illness (BP): the CGI-BP Psychiatry Res 1997 73 159 171 10.1016/S0165-1781(97)00123-6 9481807

[B18] Trivedi MH Rush AJ Ibrahim HM Carmody TJ Biggs MM Suppes T Crismon ML Shores-Wilson K Toprac MG Dennehy EB Witte B Kashner TM The Inventory of Depressive Symptomatology, Clinician Rating (IDS-C) and Self-Report (IDS-SR), and the Quick Inventory of Depressive Symptomatology, Clinician Rating (QIDS-C) and Self-Report (QIDS-SR) in public sector patients with mood disorders: a psychometric evaluation Psychol Med 2004 34 73 82 10.1017/S0033291703001107 14971628

[B19] Altman EG Hedeker D Peterson JL Davis JM The Altman Self-rating Mania Scale Biol Psychiatry 1997 42 948 955 10.1016/S0006-3223(96)00548-3 9359982

[B20] Trompenaars FJ Masthoff ED Van Heck GL Hodiamont PP De Vries J Content validity, construct validity, and reliability of the WHOQOL-Bref in a population of Dutch adult psychiatric outpatients Qual Life Res 2005 14 151 160 10.1007/s11136-004-0787-x 15789949

[B21] Rosa AR Sanchez-Moreno J Martinez-Aran A Salamero M Torrent C Reinares M Comes M Colom F Van Riel W Ayuso-Mateos JL Kapczinski F Vieta E Validity and reliability of the Functioning Assessment Short Test (FAST) in bipolar disorder Clin Pract Epidemiol Ment Health 2007 3 5 10.1186/1745-0179-3-5 17555558 PMC1904447

[B22] Hogan TP Awad AG Eastwood R A self-report scale predictive of drug compliance in schizophrenics: reliability and discriminative validity Psychol Med 1983 13 177 183 10.1017/S0033291700050182 6133297

[B23] van Wijngaarden B Schene AH Koeter M Vazquez-Barquero JL Knudsen HC Lasalvia A McCrone P Caregiving in schizophrenia: development, internal consistency and reliability of the involvement evaluation questionnaire--european version. EPSILON study 4. European psychiatric services: inputs linked to outcome domains and needs Br J Psychiatry Suppl 2000 39 s21 s27 10945074 10.1192/bjp.177.39.s21

[B24] Hofman A Honig A Vossen M Het manisch depressief syndroom; psycho-educatie als onderdeel van behandeling Tijdschr Psychiatr 1992 34 549 559

[B25] Vieta E Blasco-Colmenares E Figueira ML Langosch JM Moreno-Manzanaro M Medina E Group WA-bS Clinical management and burden of bipolar disorder: a multinational longitudinal study (WAVE-bd study) BMC Psychiatry 2011 11 58 10.1186/1471-244X-11-58 21481244 PMC3087674

[B26] Leverich GS Nolen WA Rush AJ McElroy SL Keck PE Denicoff KD Suppes T Altshuler LL Kupka R Kramlinger KG Post RM The Stanley Foundation Bipolar Treatment Outcome Network. I. Longitudinal methodology J Affect Disord 2001 67 33 44 10.1016/S0165-0327(01)00430-X 11869751

[B27] Suppes T Leverich GS Keck PE Nolen WA Denicoff KD Altshuler LL McElroy SL Rush AJ Kupka R Frye MA Bickel M Post RM The Stanley Foundation Bipolar Treatment Outcome Network. II. Demographics and illness characteristics of the first 261 patients J Affect Disord 2001 67 45 59 10.1016/S0165-0327(01)00432-3 11869752

